# Multifaceted glycoadjuvant@AuNPs inhibits tumor metastasis through promoting T cell activation and remodeling tumor microenvironment

**DOI:** 10.1186/s12951-021-01129-3

**Published:** 2021-11-18

**Authors:** Xiaojing Xu, Minfeng Gan, Youzhen Ge, Cheng Yi, Tianyun Feng, Mengjie Liu, Cenhao Wu, Xiang Chen, Weidong Zhang, Lixiang Zhao, Jun Zou

**Affiliations:** 1 College of Basic Medicine and Biological Sciences, Medical Department, Soochow University, 215123 Suzhou, People’s Republic of China; 2 Department of Orthopaedic Surgery, The First Affiliated Hospital of Soochow University, Suzhou, 215006 Jiangsu China; 3 Institute of Blood and Marrow Transplantation, Department of Hematology, Collaborative Innovation Center of Hematology, The First Affiliated Hospital of Soochow University, Suzhou, People’s Republic of China; 4 Center for Soft Condensed Matter Physics and Interdisciplinary Research, Soochow University, Suzhou, 215006 People’s Republic of China; 5 Jiangsu Key Lab of Zoonosis/Jiangsu Co-Innovation Center for Prevention and Control of Important Animal Infectious Diseases and Zoonoses, Yangzhou University, Yangzhou, China

## Abstract

**Background:**

Cytosine-phosphate-guanine (CpG) dinucleotides has been used as adjuvants for cancer immunotherapy. However, unmodified CpG are not very efficient in clinical trials. Glucose, ligand of C-type lectin receptors (CLRs), can promote DC maturation and antigen presentation, which is the first step of induction of adaptive immune responses. Therefore, conjugation of type B CpG DNA to glucose-containing glycopolymers may enhance the therapeutic effects against tumor by CpG-based vaccine.

**Methods:**

gCpG was developed by chemical conjugation of type B CpG DNA to glucose-containing glycopolymers. The therapeutic effects of gCpG-based vaccine were tested in both murine primary melanoma model and its metastasis model.

**Results:**

gCpG based tumor vaccine inhibited both primary and metastasis of melanoma in mice which was dependent on CD8 + T cells and IFNγ. In tumor microenvironment, gCpG treatment increased Th1 and CTL infiltration, increased M1 macrophages, decreased Tregs and MDSCs populations, and promoted inflammatory milieu with enhanced secretion of IFNγ and TNFα. The anti-tumor efficacy of gCpG was dramatically enhanced when combined with anti-PD1 immunotherapy.

**Conclusions:**

We confirmed that gCpG was a promising adjuvant for vaccine formulation by activating both tumor-specific Th1 and Tc1 responses, and regulating tumor microenvironments.

**Graphical Abstract:**

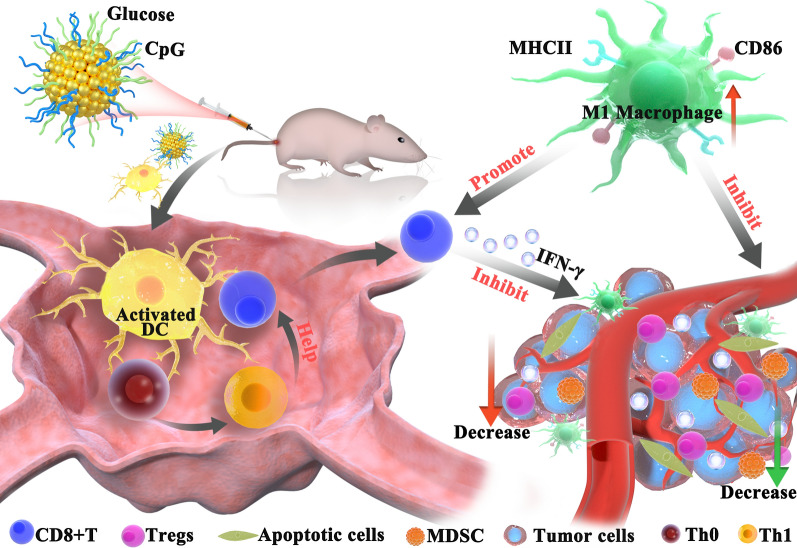

## Background

Cytotoxic T lymphocyte (CTL) is a key player of anti-tumor immune response [1–3]. Toll-like receptor (TLR) agonists have been widely tested as vaccine adjuvants, since they can stimulate innate immune response, followed by adaptive immune response [4]. Unmethylated CpG, agonist of TLR9, activates type I immunity and has been widely used as adjuvant to stimulate CTLs. However, unmodified CpG-based tumor vaccines are not very efficient in clinical trials [10]. The main reasons of the failure of unmodified CpG-based tumor vaccines are those: (1) unmodified CpG stimulates relatively weak CTL response in vivo, and (2) in tumor microenvironment (TME), CTL is always inhibited by immune suppressive cells, including tumor-associated macrophages (TAMs), regulatory T cells (Tregs) and myeloid-derived suppressor cells (MDSCs) [11–13].

Carbohydrates play the most important role in living bodies because which can be used as food for energy sources, and as ligand for selectively binding with protein through recognition of C-type lectin receptors (CLRs) [14, 15]. CLRs signal is important for antigen presentation on DCs maturation, which further induce adaptive immunity. For instance, the high level of glucose can promote DCs maturation [16]. On the contrary, lack of glucose dampens antitumor activities of tumor-infiltrating T cells in tumor microenvironment [17]. Therefore, CLR-targeted (glucose) and TLR9-targeted (CpG) glycopolymer-based adjuvant may have powerful therapeutic effects against cancer.

In present study, glycoadjuvant@AuNPs (gCpG) were obtained by conjugating CpG to glucose-containing glycopolymers [18], and the therapeutic effects of gCpG-based tumor vaccine were tested on melanoma metastasis model. We found that gCpG based tumor vaccine controlled both primary tumor and its metastasis. It promoted DC maturation, enhanced the antigen-specific CTL response and regulated tumor microenvironments by decreasing Tregs and MDSCs, and promoting polarization towards M1 macrophages, which may be used for cancer vaccine formulation.

## Materials and methods

### Glycoadjuvant@AuNPs (gCpG) preparation

The glycoadjuvant@AuNPs (gCpG) could be obtained using our reported method. First, glycopolymer could be successfully prepared by photo-induced reversible addition fragmentation chain transfer (RAFT) polymerization using 2-cyanoprop-2-yl-α-dithionaphthalate (CPDN) as RAFT agent, 2-(methacrylamido) glucopyranose (MAG), and N-3,4-dihydroxybenzenethyl methacrylamide (DMA) as monomer. Then, the catechol-containing glycopolymer was employed to prepare glycoadjuvant@AuNPs in one pot, where catechol-containing glycopolymer, HAuCl_4_, and amine-functionalized CpG (CpG-NH_2_) could react in a single step to form adjuvant.

### In vitro experiments

#### Cell lines

B16-OVA cells were obtained from ATCC and maintained in complete DMEM medium (Gibco) with 10% fetal bovine serum.

#### Immune cell preparation

The homogenate of spleens and tumor tissues were filtered through 70-mm nylon meshes, and single-cell suspensions of the immune cells were obtained after separation on a Percoll gradient of 40% density. EasySep™ Mouse CD8^+^ T Cell Isolation Kit (Stemcell) was used to isolate CD8^+^ T cells from splenocytes.

#### Proliferation assay of T cells

After 24 h co-culturing with OVA protein, splenocytes were irradiated with 200 Gy via a ^60^Go source, and used as stimulatory cells. Stimulatory cells were added to 5.5 × 10^3^ CD4^+^ or 5 × 10^3^ CD8^+^ T cells. After about 64 h co-culture, CCK8 was added to the co-cultured system in a final concentration of 10%. After 2 h incubation, OD450 values were detected and used for the counting of stimulation index (SI). The following forma were used to calculate SI: (OD450 values of T cells co-cultured with OVA pulsed stimulatory cells  − OD450 values of media control)/(OD450 values of T cells co-cultured with unstimulated splenocyte  − OD450 values of media control).

#### Tumor-killing assay

Effectors in this assay were CD8^+^ T or splenocytes without CD8^+^ T cells. Targets (4 × 10^3^ B16-OVA) were plated and incubated for 2 h in 96-well plate. Then effectors were added to and co-cultured with targets at different ratios. Tumor-killing was detected by using CytoTox 96^®^ Non-Radioactive Cytotoxicity Assay purchased from Promega. The percentages of tumor-killing were calculated: (experimental  − targets spontaneous-effectors spontaneous) × 100/(targets maximum  − targets spontaneous).

#### Bone marrow DC (BMDC) generation

BMDC were collected from the femur and tibia of mice and cultured in RPMI 1640 medium supplemented with GM-CSF (10 ng/mL), IL-4 (10 ng/mL) and FCS (10%). The medium was half-replaced every 2 days, and BMDCs were harvested after 7 days.

#### Flow cytometry assay

The following monoclonal antibodies (BD Biosciences and Biolegend) were used: FITC-anti CD4 (RM4-5), FITC-anti NK1.1 (PK136), FITC-anti CD19 (1D3), FITC-anti CD62L (MEL-14), FITC-anti MHC II (M5), FITC-anti F4/80 (BM8), PE-antiCD3e (145-2C11), PE-anti IL4 (11B11), PE-anti IFNγ (XMG1.2), PE-anti CD206 (C068C2), PE-anti Gr1 (RB6-8C5), PE-anti CD44 (IM7), PE-anti CD16/32 (93), PerCP Cy5.5-anti CD8a (53–6.7), PerCP Cy5.5-anti Gr1 (RB6-8C5), PE-Cy7-anti CD11b (M1/70), PE-Cy7-anti CD11c (HL3), APC-anti CD45 (30-F11), APC-anti Foxp3 (150D), APC-anti PD1 (29F.1A12) and APC-anti TNFα (MP6-XT22). Single-cell suspensions was staining at 4 °C with antibodies in PBS supplemented with 1% BSA, 0.1% NaN_3_, 0.2 mM EDTA and Fc-blocker for about 30 min. The results were analyzed with FACS CantoII (BD Biosciences) using CellQuest software.

#### OVA-specific CD8^+^ T staining assay

TILs were stained with OVA-specific tetramers (Beckman Coulter), Fc-blocker and APC-anti CD8 at room temperature. 30 min later, TILs were washed and resuspended in PBS supplemented with 1% BSA, 0.1% NaN_3_ and 0.2 mM EDTA, and analyzed with FACS CantoII (BD Biosciences).

### In vivo experiments

#### Murine melanoma therapeutic models

Animal experiments were approved by the Animal Experiment Ethics Committee of Soochow University, and performed under specific-pathogen-free (SPF) conditions. 6-week-old SPF female C57BL/6 mice were purchased from the Shanghai Laboratory Animal Center (Shanghai, China). Animals were euthanized in a CO_2_–containing chamber, at the end of each experiment.

Primary melanoma models were generated by *s.c.* injection of 2.5 × 10^5^ B16-OVA cells into the left flank of the animal. At day 7, vaccines were *s.c.* immunized at the right flank, and the immunization was repeated at day 12. From day 9, the longest dimension (L) and the shortest dimension (width, W) of the tumors were daily measured. Tumor volume were calculated by L × W^2^/2.

Metastasis models were generated by *i.v.* injection of 9 × 10^5^ B16-OVA from tail vein of each mouse. At day 7 and 12, vaccines were *s.c.* immunized at the right flank, respectively. Animals were sacrificed at day 19, and the lung metastatic nodules were counted.

#### Immunohistochemistry and histopathology

Paraffin tumor sections were sequentially deparaffinized. After overnight incubation with anti-CD4 (GK1.5, Abcam) or anti-CD8 (2.43, Abcam) antibodies, HRP-conjugated rabbit anti-rat IgG antibodies were added. After nuclear counter-staining with hematoxylin (Solarbio), the presences of CD4 or CD8 protein were visualized by DAB staining.

#### Adoptive transferring CD8^+^ T cells

Mouse was inoculated with 9 × 10^5^ B16-OVA cells from tail vein, and splenic CD8^+^ T cells were separated 7 days later. About 4 × 10^6^ isolated CD8^+^ T cells were injected *i.v.* into mice with metastasis. The lung metastatic nodules were examined 12 days later.

#### In vivo cell depletion

Antibody specific to mouse CD4 (GK1.5), CD8 (53–6.7), Gr1 (RB6-8C5) and IFN-γ (XMG1.2) purchased from Bioxcell were used to deplete CD4^+^, CD8^+^ cells, MDSC, and neutralize IFN-γ, respectively. Mouse was *i.p.* injected with appropriate antibodies (200 µg per dose) 1 day before vaccines immunization, and the antibodies with the same dose were repeatedly inoculated 7 days later. Clophosome (Anionic Liposomal Clodronate, FormuMax) was used to deplete macrophages by *i.p.* injection of 800 µg 2 days before and 5 days after the first vaccination. The efficacy of cell depletion was confirmed by flow cytometric analysis.

### Statistical analysis

Each experiment was triplicate performed. Data were expressed as mean ± standard deviation (SD). The statistically significant difference between groups was evaluated by GraphPad Prism 8 software for Windows (GraphPad Software, San Diego, CA). *p* < 0.05 was considered to be a significant difference. The significance levels are marked *p < 0.05, **p < 0.01, ***p < 0.001 and ****p < 0.0001.

## Results

### gCpG inhibits tumor growth and promotes T cell responses in primary melanoma

**Fig. 1:**
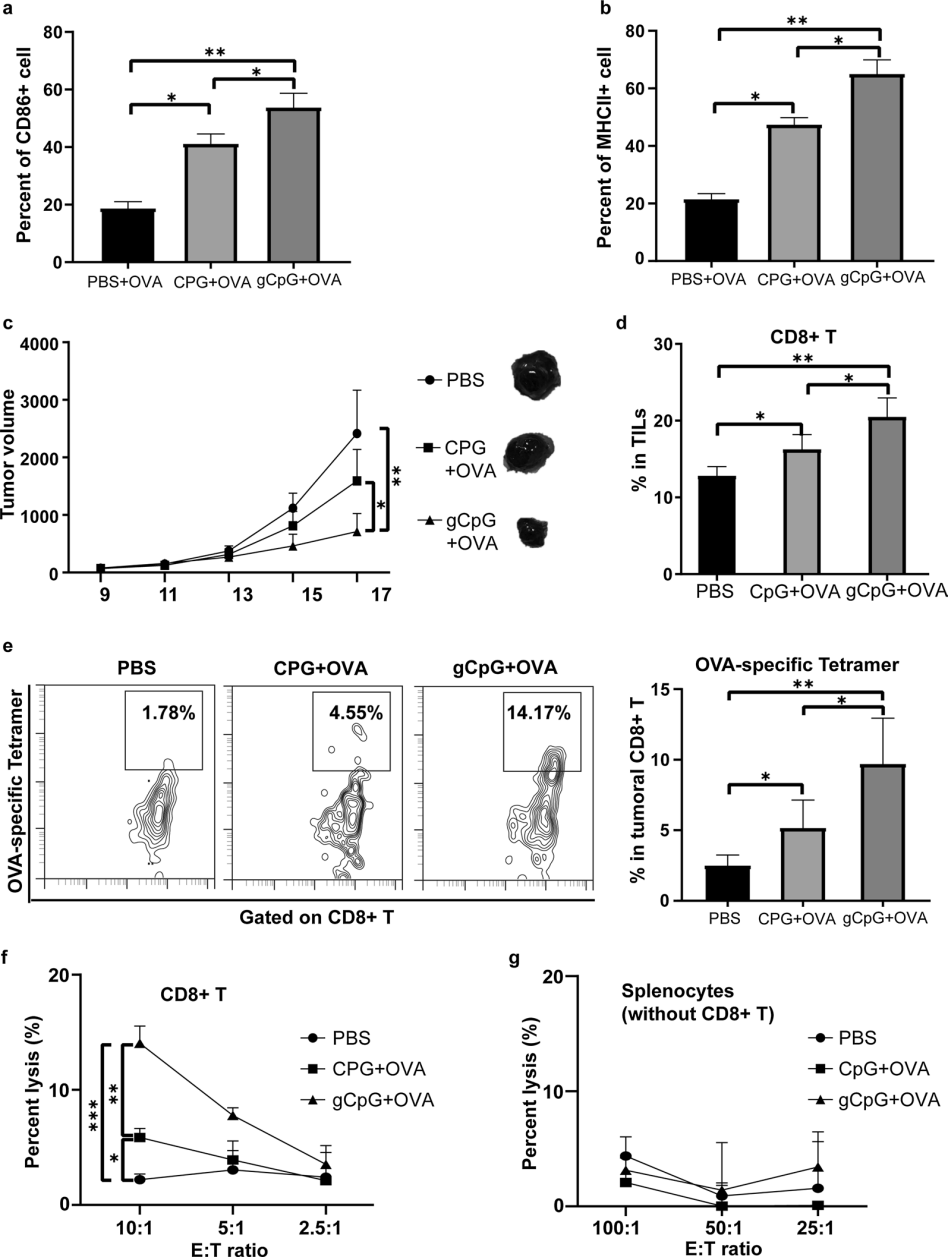
gCpG inhibits tumor growth and promotes T cell responses in primary tumor model. The levels of CD86 (**a**) and MHC II (**b**) expression on BMDC were measured by flow cytometry. **c** Tumor growth of melanoma-bearing mice. Mice were injected with 2 × 10^5^ B16-OVA cells, and immunized s.c. with one of the three tumor vaccines at the left flank on day 7 and 12 post tumor inoculation, respectively. **d** Mice were sacrificed on day 19 after tumor inoculation. Percentage of tumor-infiltrating CD8^+^ T cells were analyzed by flow cytometry. **e** Tetramer staining of OVA–specific CD8^+^ T cells in TILs. Cytotoxicity assay of splenic CD8^+^ T cells (**f**) and other population without CD8^+^ T cells (**g**) from the tumor-bearing mice against B16-OVA tumor cells. The specific killings were determined using CytoTox 96 nonradioactive cytotoxicity assay. The data shown are the representative of at least three experiments. *p < 0.05 and **p < 0.01, ***p < 0.001 and ****p < 0.0001

gCpG can enhance the expression of secreted embryonic alkaline phosphatase (SEAP) by macrophages [18], indicating that glycopolymer-modification can improve the CpG’s immune stimulatory activity. To examine the effects of gCpG on DC maturation, BMDCs were cocultured with PBS, CpG (2 µg/mL) or gCpG (2 µg/mL), and the surface markers (CD86 and MHC II) were examined by FACs. The results showed that gCpG promoted BMDC maturation by upregulating expression of MHC II and CD86 molecules (Fig. 1a and b).

To determine the therapeutic effects of gCpG againt primary tumor, mice bearing primary melanoma were immunized with each vaccine, respectively. gCpG + OVA treatment effectively controlled tumor growth (Fig. 1c), whereas CpG + OVA treatment only slightly inhibited tumor growth.

Cell populations in tumor were examined on day 19 post tumor injection using flow cytometry. gCpG + OVA treatment increased the CD8^+^ T prevalence in tumor sites (Fig. 1d). Tetramer staining assay further revealed that gCpG + OVA treatment significantly enhanced OVA-specific CD8^+^ T cells infiltrating into tumor site (Fig. 1e). gCpG + OVA treatment greatly enhanced CD8^+^ T killing capacity against B16-OVA cells (Fig. 1f), while had no obvious effects on other populations killing capacity (Fig. 1g). Together, above results revealed that gCpG based tumor vaccine could inhibit primary melanoma and promote CD8^+^ T cell responses.

### gCpG inhibits lung metastasis of melanoma and promotes T cell responses

**Fig. 2:**
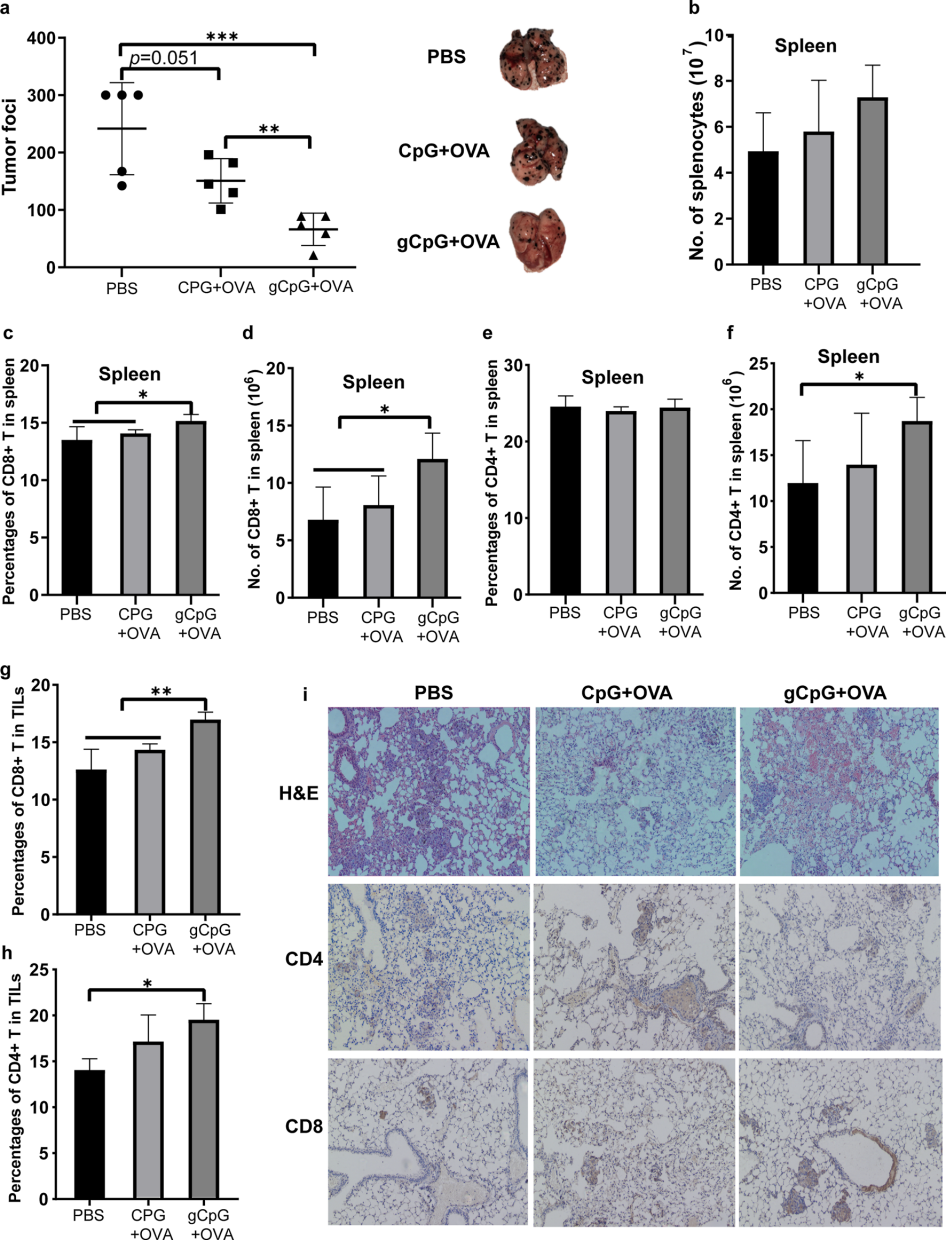
gCpG inhibits lung metastasis of melanoma. **a** The numbers of metastatic nodules in the lungs of the tumor-bearing mice. Mice were injected i.v. with 8 × 10^5^ B16-OVA from tail vein. At day 7 and 12 post tumor inoculation, mice were immunized with different vaccines. Seven days later, mice were sacrificed, and the metastatic nodules in lungs were quantified. Total cell numbers of splenocytes (**b**), frequencies and total cell numbers of CD8^+^ T (**c** and **d**), and CD4^+^ T (**e** and** f**) cells in the spleen and frequencies of tumor-infiltrating CD8^+^ T (**g**) and CD4^+^ T cells (**h**) were analyzed by flow cytometry. **i** H&E and immunochemical staining for CD4^+^ and CD8^+^ T cells of tumor tissues from melanoma-bearing mice (200 × magnification). The assays were done in quadruplicates. The data shown are the representative of at least three experiments. *p < 0.05 and **p < 0.01, ***p < 0.001 and ****p < 0.0001

Metastasis is considered to be the leading cause of death for patients with cancer. Therefore, we wondered if gCpG included vaccine may also control melanoma metastasis. Animals were *i.v.* inoculated with B16-OVA, and vaccines were given at 7- and 12-days post tumor injection. gCpG + OVA significantly reduced the metastatic nodes of melanoma in lung (Fig. 2a), while CpG + OVA only exhibited slightly inhibition of tumor metastases.

To examine possible mechanisms of therapeutic efficacy of gCpG included vaccine, splenocytes and TILs were analyzed. gCpG + OVA treatment didn’t significantly change the total numbers of splenocytes (Fig. 2b). CD8^+^ T prevalence and total numbers were significantly increased in spleen after gCpG + OVA treatment (Fig. 2c and d). Although the percentages of CD4^+^ T cells were similar in spleen among each group, the CD4^+^ T numbers were significantly increased after CpG + OVA treatment (Fig. 2e and f).

After gCpG + OVA treatment, tumoral infiltration of CD8^+^ T was significantly increased (*p* < 0.01, Fig. 2g). gCpG + OVA treatment also significantly increased CD4^+^ infiltration, as compared with PBS treatment (Fig. 2h). CD8^+^ and CD4^+^ T cells infiltrations in tumor sites were also confirmed by IHC (Fig. 2i).

### gCpG promotes antigen-specific Th1 cytokines secretion by both CD4^+^ and CD8^+^ T cells

**Fig. 3:**
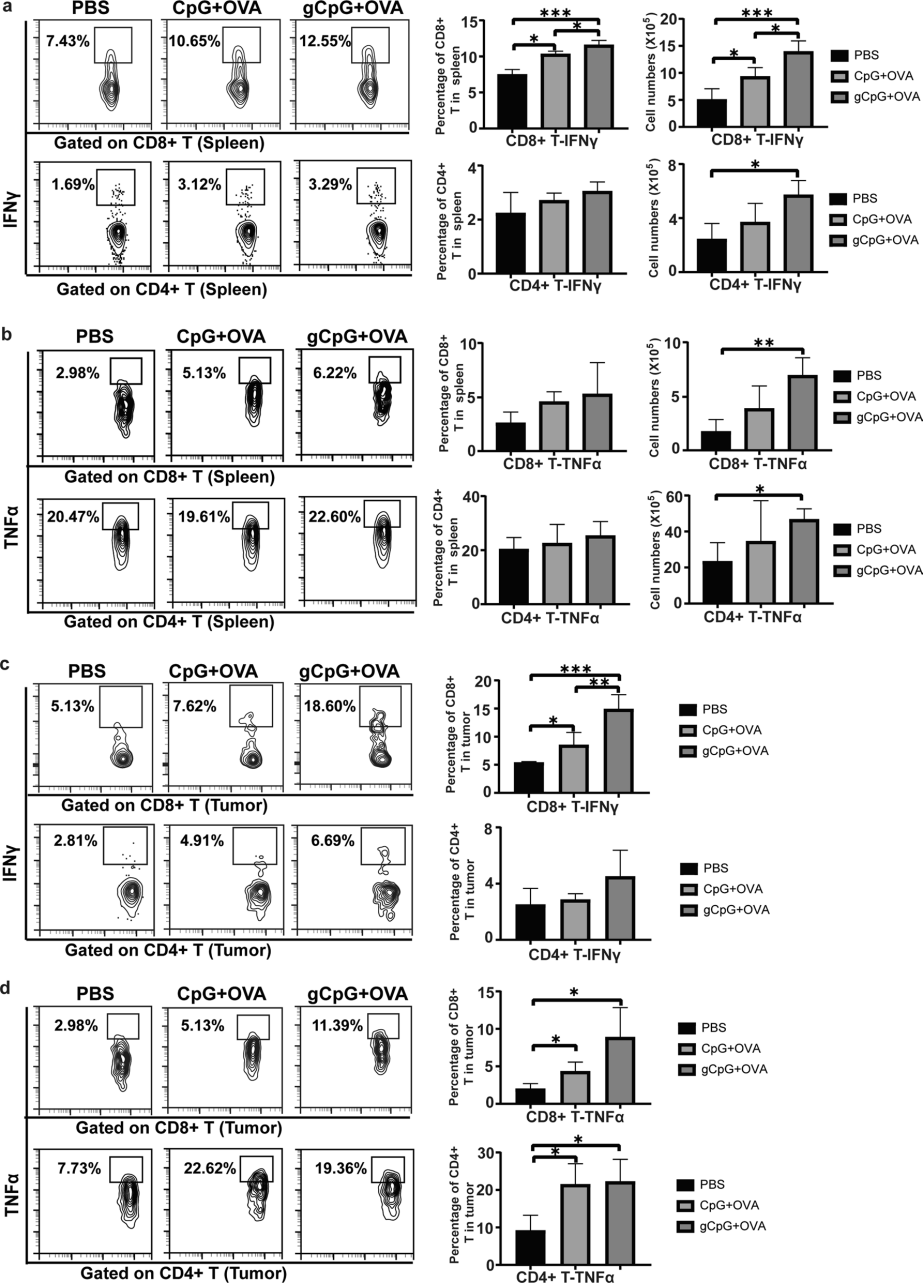
gCpG promotes tumor specific Th1 cytokines production on CD4^+^ and CD8^+^ T cells. **a** Intracellular staining of IFNγ production on CD8^+^ and CD4^+^ T cells from splenocytes stimulated with OVA. **b** Intracellular staining of TNFα expression on CD8^+^ and CD4^+^ T cells from splenocytes stimulated with OVA. **c** Intracellular staining of IFNγ production on CD8^+^ and CD4^+^ T cells from TILs stimulated with OVA. **d** Intracellular staining of TNFα expression on CD8^+^ and CD4^+^ T cells from TILs stimulated with OVA. The assays were done in quadruplicates. The data shown are the representative of three experiments. *p < 0.05 and **p < 0.01, ***p < 0.001 and ****p < 0.0001

Intracellular staining was used to examine the antigen-specific T-cell in melanoma metastasis model. gCpG + OVA treatment significantly enhanced the percentage and total numbers of IFNγ^+^CD8^+^ T in spleen, compared to PBS treatment (*p* < 0.0001, Fig. 3A). Although different treatments didn’t significantly change the percentage of IFNγ-producing CD4^+^ T cells in spleen, gCpG + OVA treatment significantly increased the total cell numbers of this population (*p* < 0.05, Fig. 3a). The total numbers of TNFα^+^CD8^+^ T cells and TNFα^+^CD4^+^ T cells in spleen were also increased after gCpG + OVA treatment (Fig. 3b).

In TILs, gCpG + OVA treatment significantly enhanced IFNγ (*p* < 0.0001, Fig. 3c) and TNFα (*p* < 0.05, Fig. 3d) production in tumoral infiltrating CD8^+^ T cells. Besides, CpG + OVA also significantly enhanced the percentages of IFNγ^+^CD4^+^ and TNFα^+^CD4^+^ T, as compared with PBS treatment (Fig. 3c and d). These results indicated that gCpG + OVA promoted both Tc1 and Th1 responses in metastatic melanoma model.

### gCpG promotes antigen-specific CTL response

**Fig. 4:**
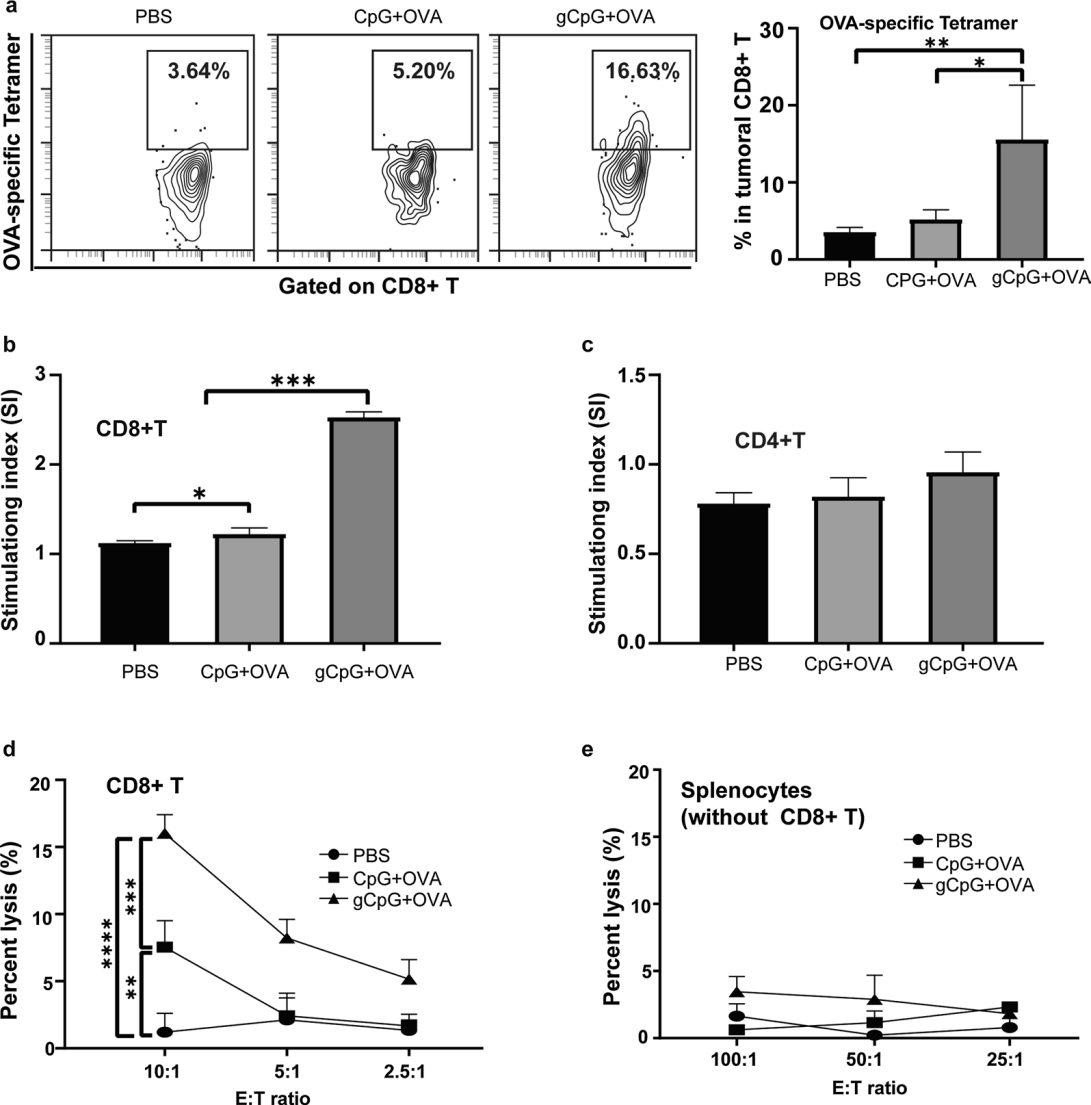
gCpG promotes antigen-specific CD8^+^ T cells response. **a** Tetramer staining of OVA–specific CD8^+^ T cells in TILs. Proliferations of CD4^+^ (**b**) or CD8^+^ (**c**) T cells isolated from spleen were determined using CCK8 cell counting kits. The stimulation index (SI) is calculated as the ratio of the proliferation of cells received OVA-specific stimulation to cells without OVA-specific stimulation in the same group. Cytotoxicity assay of splenic CD8^+^ T cells (**d**) and other population without CD8^+^ T cells (**e**) from the tumor-bearing mice against B16-OVA tumor cells. The specific killings were determined using CytoTox 96 nonradioactive cytotoxicity assay. *p < 0.05 and **p < 0.01, ***p < 0.001 and ****p < 0.0001

CD8^+^ T is one of most powerful effector cells of anti-tumor immune responses. First, we detected tumor specific CD8^+^ T population by tetramer staining assay, and the results showed that gCpG + OVA treatment significantly increased tumoral infiltration of OVA-specific CD8^+^ T (Fig. 4a). gCpG + OVA treatment also greatly enhanced the proliferation of CD8^+^ T (*p* < 0.0001, Fig. 4b), while vaccine treatments didn’t promote the proliferation of CD4^+^ T cell (Fig. 4c). gCpG + OVA also dramatically enhanced CD8^+^ T killing capacity (*p* < 0.00001, Fig. 4f), while splenocytes excluded with CD8^+^ T couldn’t kill tumor cells directly (Fig. 4d and e). These data indicated that gCpG + OVA promoted functions of antigen-specific CD8^+^ T cell.

### Antigen-specific CD8^+^ T and IFNγ are critical for gCpG treatment

**Fig. 5:**
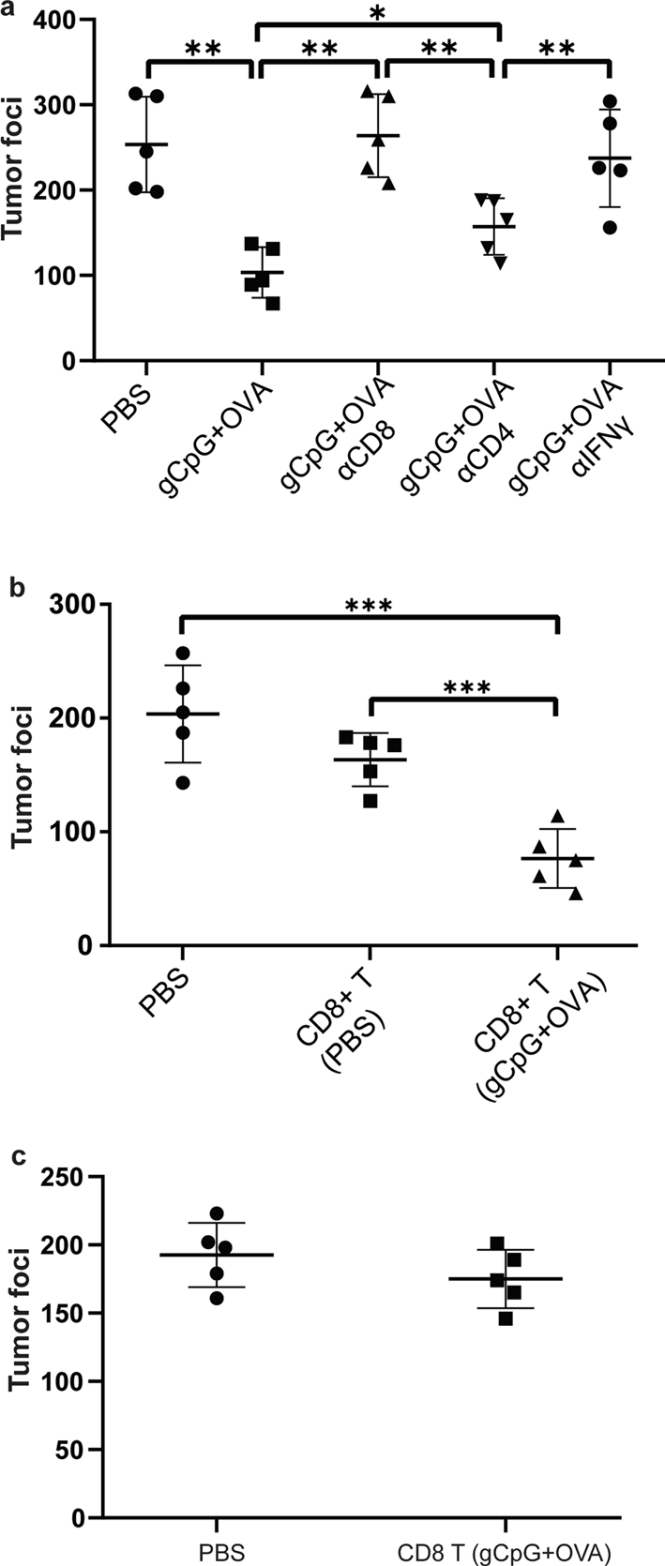
Antigen-specific CD8^+^ T cells are critical for gCpG treatment. **a** Tumor foci of vaccine-treated mice were injected i.p. with 1 mg GK1.5 (anti-mouse CD4 mAb) or 53–6.7 (anti-mouse CD8 mAb) 2 days before the first immunization, and the injections were repeated 7 days later. Mice were sacrificed on day 7 post the last immunization and the metastatic nodules were counted. **b** 8 × 10^5^ B16-OVA were i.v. injected into each mouse from tail vein. On day 7, 3 × 10^6^ CD8^+^ T cells isolated from spleen of tumor-bearing mice treated with PBS or gCpG + OVA were i.v. injected into tumor-bearing mice. Mice were sacrificed and the metastatic nodules in lung were counted 12 days later. **c** Mice with B16-F10 metastasis were adoptively transferred with 3 × 10^6^ T cells from gCpG + OVA treated mice. Mice were sacrificed and the metastatic nodules in lung were counted 12 days later. *p < 0.05 and **p < 0.01, ***p < 0.001 and ****p < 0.0001

Th1 and CTL cells are important effectors cells for anti-tumor response and we observed the strong activation of both Th1 and CTL upon gCpG + OVA treatment. In order to examine which cell type plays the critical role in the anti-tumor response, CD4^+^ and CD8^+^ T cells were depleted by specific antibodies in metastasis model, respectively. Anti-tumor effects of gCpG based vaccine were partially reduced as the lack of CD4^+^ T cells, while depletion of CD8^+^ T abrogated the anti-tumor efficacy of the vaccine (Fig. 5a), indicating that gCpG based vaccine controlled tumor growth by CD8^+^ T cells. Adoptive transferring CD8^+^ T cells from mice treated with gCpG + OVA inhibited melanoma metastasis, on the contrary CD8^+^ T from PBS treated group had no therapeutic effects (Fig. 5b), confirmed the critical role of CD8^+^ T in controlling tumor metastasis by gCpG + OVA. IFN-γ is a key molecule produced by effector CD8^+^ T cells. IFN-γ neutralization almost abrogated gCpG efficacy, indicated that the therapeutic effects of gCpG depended on IFN-γ derived from antigen-specific CD8^+^ T (Fig. 5a).

To examine whether the gCpG + OVA induced CD8^+^ T response was limited against OVA, mice with B16-F10 metastasis were adoptively transferred with CD8^+^ T cells isolated from mice treated with gCpG + OVA. The results showed that CD8^+^ T cells from mice treated with gCpG + OVA couldn’t inhibit B16-F10 melanoma, indicated that gCpG + OVA induced CD8^+^ T cell response was OVA-specific (Fig. 5c).

### gCpG regulates tumor microenvironment

**Fig. 6:**
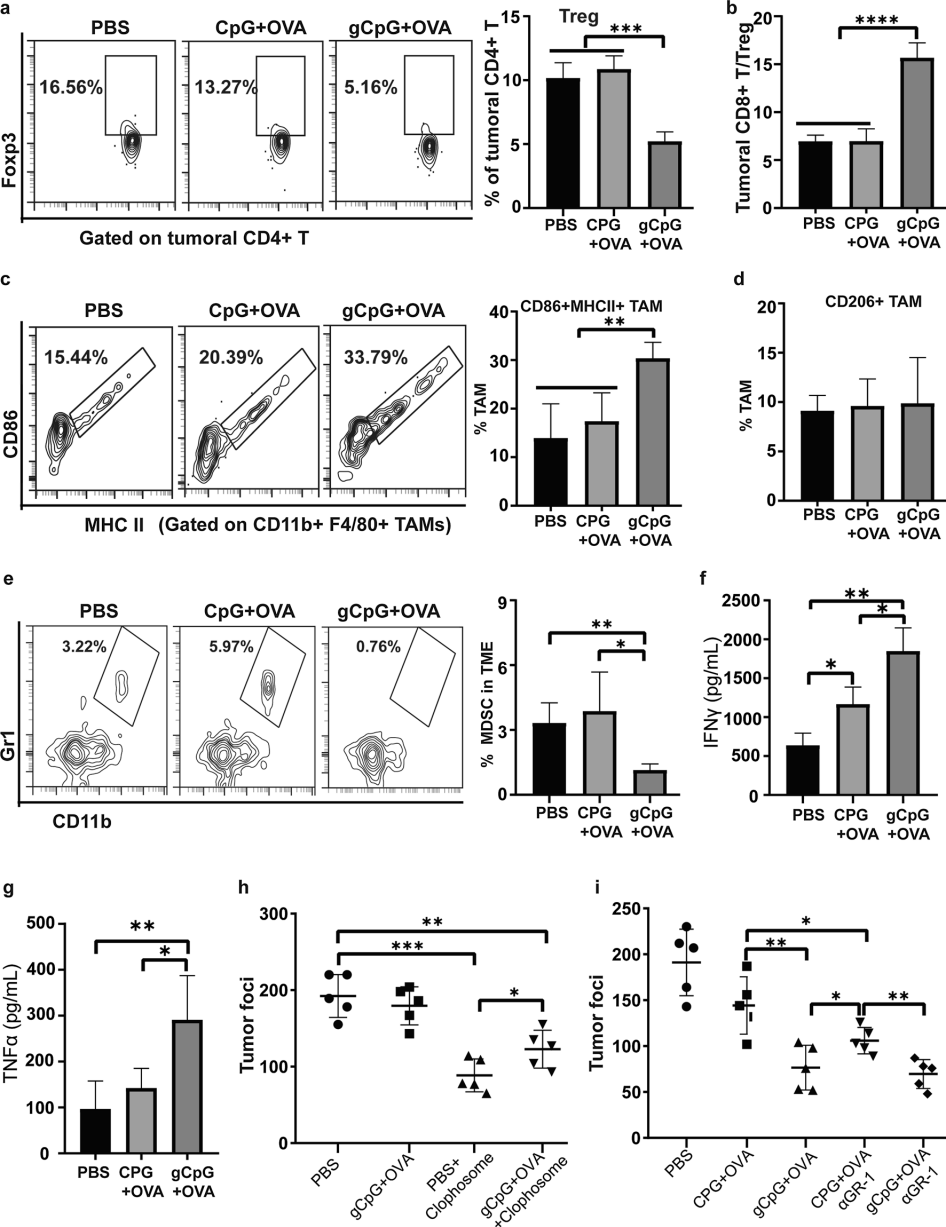
gCpG regulates tumor microenvironment. Tumor-bearing mice were immunized with PBS, CpG + OVA or gCpG on day 7 and 12. On day 19, **a** frequency of Tregs, **b** CD8/Treg ratio, percentages of both M1 macrophages (**c**) and M2 macrophages (**d**), and prevalence of CD11b^+^Gr1^+^ MDSCs cells (**e**) in the TME were determined by FACS. **f** and **g** IFNγ and TNFα level in supernatants of tumor. **h** and** i** Tumor foci of vaccine-treated mice with depletion of macrophages or MDSCs by i.p. injection of 800 µg Clophosome or 250 μg anti-Gr-1 antibody 2 day before vaccination, and the injections were repeated 7 days later. Mice were sacrificed on day 7 post the last immunization and the metastatic nodules were counted. The experiments were performed with 5–7 mice per group. The assays were done in quadruplicates. The data shown are the representative of three experiments. *p < 0.05 and **p < 0.01, ***p < 0.001 and ****p < 0.0001

T cell function can be dampened by immunosuppressive cell populations existing in tumor microenvironment such as Tregs, TAMs and MDSCs. In present study, gCpG + OVA treatment significantly decreased the percentages of Tregs (Fig. 6a), combined with increased CD8^+^ T population, which dramatically increased CD8^+^ T/Treg ratio in gCpG + OVA treated mice (Fig. 6b).

TAMs contained two phenotypes, the M1 phenotype (CD86^+^ MHCII^+^) and M2 phenotype (CD206^+^). The former phenotype promotes anti-tumor responses, while the later exerts immunosuppressive functions. gCpG + OVA treatment significantly increased M1 TAMs in TME (Fig. 6c), but had no effects on M2 TAMs (Fig. 6d). MDSCs are very potent suppressors of cytotoxic T-cell immunity. gCpG + OVA treatment dramatically decreased the infiltration of Gr1^+^CD11b^+^ MDSCs (Fig. 6e). IFNγ and TNFα (Th1 cytokine) expressions in TME were also determined by ELISA. Consistent with FACs results, gCpG treatment significantly increased IFNγ and TNFα level in TME (Fig. 6f and g), suggesting gCpG promoted inflammatory milieu in TME.

To define the role of TAMs in the progress of tumor, Clophosome was used to deplete macrophages in vivo. Depletion of TAMs significantly promoted melanoma metastasis in gCpG + OVA group, while only slightly promoted tumor metastasis in PBS group (Fig. 6h), which might be a result of high percentage of M1 TAMs infiltration in gCpG + OVA treated group.

MDSCs depletion enhanced therapeutic effects of CpG based tumor vaccine greatly, as the high proportion of MDSCs infiltration in this group. However, MDSCs depletion only slightly enhanced the therapeutic effects of gCpG based vaccine, as the very low proportion of MDSCs infiltration in this group (Fig. 6i). These results suggested that decreasing MDSCs infiltration might play an important role in the therapeutic effects induced by gCpG + OVA treatment.

### Synergetic enhancement of therapeutic efficacy by gCpG combined with anti-PD1 antibody

**Fig. 7:**
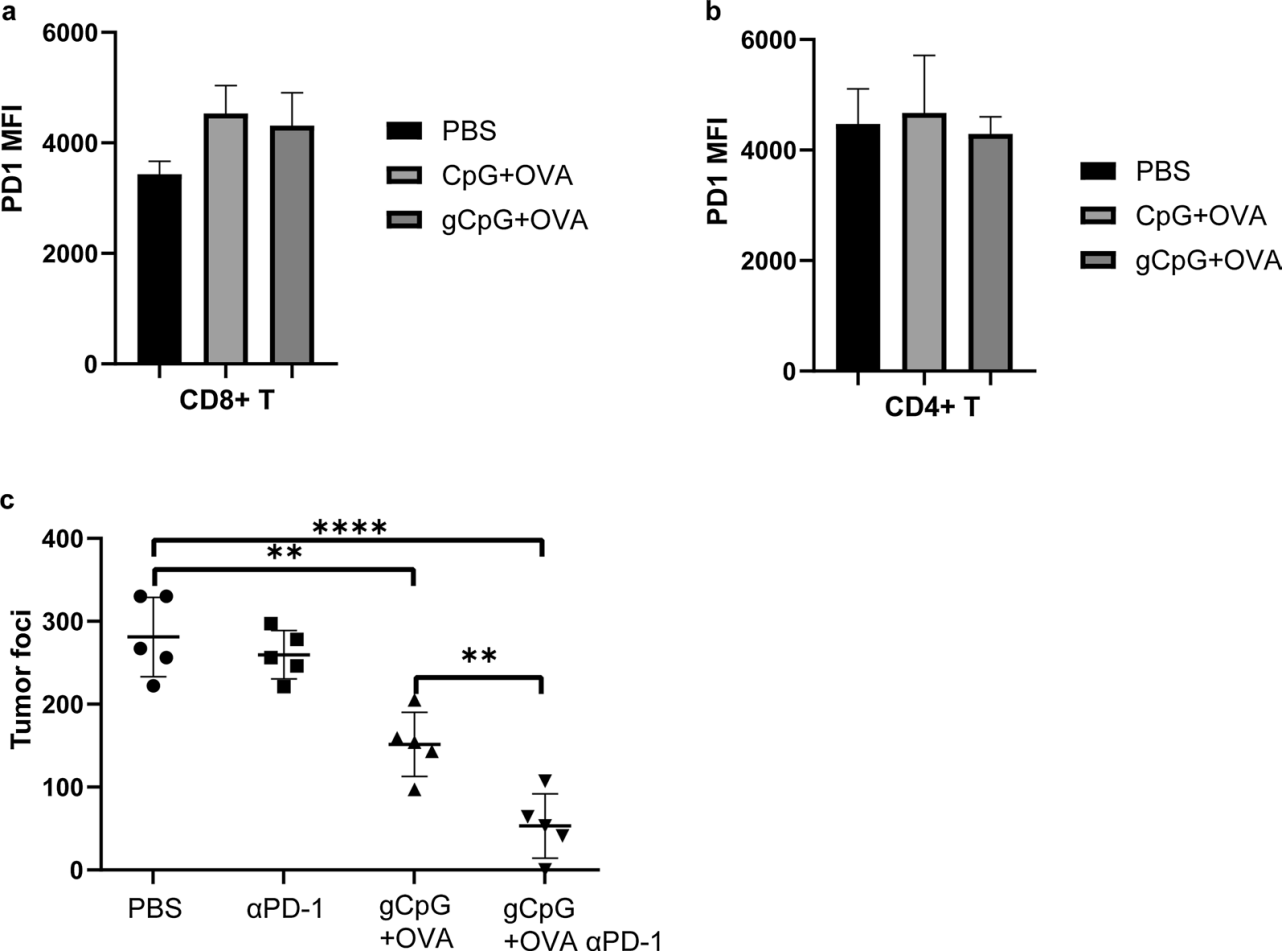
Synergetic enhancement of therapeutic efficacy by gCpG combined with anti-PD1 antibody. PD1 expression (MFI) on tumor infiltrating CD8^+^ (**a**) and CD8^+^ (**b**) T cells. **c** Metastatic nodules of 2 × 10^6^ B16-OVA mice treated with PBS, CpG + OVA or gCpG + OVA on day 17. On days 5 and 10, 200 μg α-PD1 was administered i.p. The data shown are the representative of three experiments. *p < 0.05 and **p < 0.01, ***p < 0.001 and ****p < 0.0001

Programmed cell death 1 (PD1), a coinhibitory receptor expressed on activated T cells, can inhibit the activities of tumor-infiltrating T cells in TME, and thus promote tumor progress. Targeted PD1 therapies have become commonly used to enhance T cell responses and show efficacy in multiple cancers. gCpG + OVA treatment didn’t change PD1 expression on either CD8^+^ (Fig. 7a) or CD4^+^ T cells as compared with other therapy (Fig. 7b). We wonder whether combining with anti-PD1 Ab therapy promotes the therapeutic efficacy of the gCpG included vaccine. Then, the therapeutic effect of gCpG + OVA in combination with PD1 blockade strategy was evaluated on metastasis model. gCpG + OVA vaccine in combination with anti-PD1 immunotherapy exhibited a synergetic enhancement of therapeutic effects against metastasis, while anti-PD1 therapy alone could not inhibit metastasis (Fig. 7c), indicating the great promises of such vaccine for combination with immune checkpoint therapy.

## Discussion

**Fig. 8:**
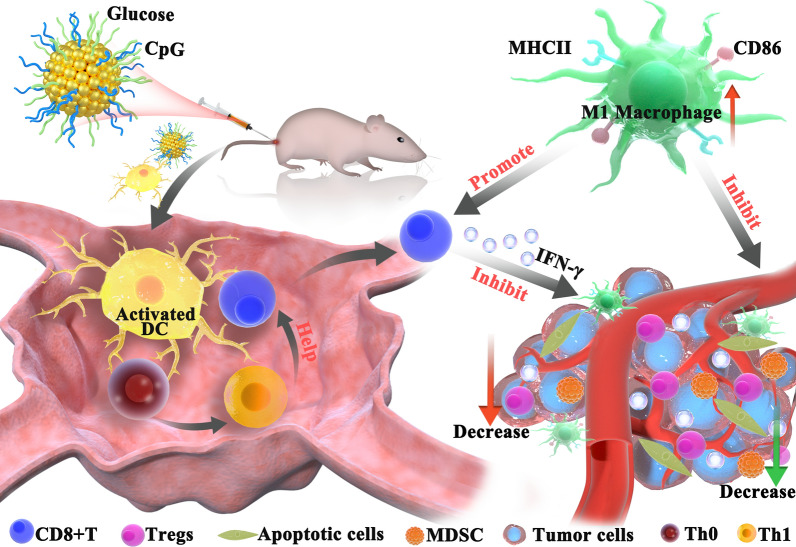
Model of gCpG action. gCpG included tumor vaccine promotes DC maturation to activate tumor-specific Th and CD8^+^ T cells. IFN-γ producing CD8^+^ T cells infiltrated the tumor. TME is also regulated by the vaccine through promoting M1 macrophage and decreasing the prevalence of Tregs and MDSCs, which promots anti-tumor immunity

Cancer vaccines are designed to activate Th1 and CTL which can mediate anti-tumor immunity. To induce efficient Th1 and CTL responses, appropriate adjuvants are required to formulate with tumor antigens. CpG is an attractive candidate to stimulate Th1 and CTL responses, which has been widely used for vaccine formulation [20]. To enhance the effects of CpG based vaccine, we conjugated type B CpG with glucose. This new adjuvant strongly improves the therapeutic effects of tumor vaccine in both primary melanoma model and metastasis model. The adjuvant activates both tumor-specific Th1 and Tc1 responses, promotes M1 macrophages polarization and proinflammatory cytokine production, and inhibits both Treg and MDSCs in TME (Fig. 8).

CTL is essential for the eradication of cancer. gCpG treatment increased CTL populations in spleen, and enhanced specific killing of CD8^+^ T against tumor. The increased CTL infiltration has been confirmed to be correlated with better clinical outcome in many cancer types, such as melanoma, lung cancer and liver cancer [21–24]. gCpG treatment greatly enhanced antigen-specific CD8^+^ T in TME, which was consistent with the therapeutic effects of the agents. Previous studies demonstrated that CpG could stimulate CD8^+^ T response, and this effect was also confirmed in present study, as CpG + OVA treatment increased killing capacity of CD8^+^ T and promoted IFNγ-producing CD8^+^ T cells infiltration in TME. Notably, glucose modification not only greatly increased the tumor-specific killing of CD8^+^ T cells, but also promoted the infiltration of TNFα- and IFNγ-producing CD8^+^ T cells in TEM. Although therapeutic effects of gCpG included vaccine were mainly dependent upon CD8^+^ T cells, Th cells were also important during the eradication of the tumor, as determined by in vivo depletion experiment. During activation by DCs, naïve CD4^+^ T cells differentiate to several effector populations, such as Th1, Th2, Th17 and Treg, according to their secretion of certain cytokines. Among them, Th1 cells, characterized by production of IFNγ and TNFα, can optimize CTL activity, and thus promote anti-tumor immunity [22–24]. On the contrary, Th2 and Treg populations promote cancer progression and its metastasis by suppressing Th1 activity [22–24]. gCpG included vaccine treatment greatly increased the production of TNFα and IFNγ in CD4^+^ T cells, whereas the productions of IL4 and IL17 in CD4^+^ T cells were hard to be detected. The potential of gCpG in driving Th1-mediate immune response, which may optimize CD8^+^ T activity, makes it a promising adjuvant for cancer immunotherapy.

Besides T cell priming, the desired cancer immunotherapy needs to overcome the immunosuppressive microenvironment in TME. However, expected antitumor efficacy was hard to achieve in clinical trials, as the high abundances of immunosuppressive cells presented in TME. Tregs is one of major suppressive populations in TME and many cancer vaccines exhibit the potential to promote and expand Tregs, resulting in suppression of antitumor immunity. We found that gCpG dramatically reduced Tregs population in TME as compared with CpG adjuvant, suggested the clinical potential of regulating Tregs in TME by gCpG. Previous studies have showed that both natural tumor progression and cancer immunotherapy outcomes in mice and humans were strongly influenced by the balance between Treg and CD8^+^ T cells in TME [25]. The high ratio of infiltrating CD8^+^ T: Tregs is often associated with prolonged survival of patient with tumor [26–28]. In present study, gCpG treatment dramatically enhanced the CD8^+^ T: Tregs ratio in TME, which is also consistent with the better therapeutic effects in metastasis model.

Macrophages play a crucial role in host defense against pathogens and in defense against tumor cells also. Macrophages can be divided into M1 and M2 in TME. It has been well documented that M1 functions in antigen-presenting activity and mediate cytotoxic functions, including anticancer activity [29]. On the contrary, M2 macrophages promote the development of tumor, and their occurrences in TME is highly correlated with poor prognosis of tumor [30]. Previous study showed that glucose upregulated CD86 expression on DC [16]. Here, we found that gCpG could promote upregulation of both CD86 and MHC II on macrophages, but the CD206 expression was not affected, suggested gCpG treatment promoted TAMs polarization toward M1 phenotype. Given the high percentage of M1 macrophages in TME and potentiating antitumor immunity of these populations, it is presumed that polarization of macrophages toward M1 in TME by gCpG plays a role in its immunotherapeutic effects on tumors. Then, depletion of macrophages in gCpG-treated mice exhibited significant progress of tumor metastasis, confirmed M1 role in controlling metastasis by gCpG. Inhibition the function of MDSCs is one of important strategies for cancer immuno-therapy [31–33]. gCpG dramatically reduced MDSCs accumulation in TME, indicated that decreasing of MDSC in TME by gCpG may play a role in its immunotherapeutic effects on tumors. Depletion of MDSC had no obvious effects in gCpG-treated mice with low prevalence of MDSCs infiltration. However, therapeutic efficacy was significantly enhanced in CpG-treated mice with high prevalence of MDSCs in TME, suggested the important role of reduction MDSCs infiltration by gCpG treatment in controlling tumor metastasis.

PD1 is one of key inhibitory molecules expressed on T cells [34, 35]. Anti-PD1 treatment alone has no obviously therapeutic effects against tumor growth, and the main reason may be the rare CD8^+^ T infiltration in tumor, especially the lack of antigen-specific CD8^+^ T. On the control, gCpG based tumor vaccine treatment dramatically enhanced tumor-infiltrating CD8^+^ T, especially antigen-specific CD8^+^ T, and the combination of anti-PD1 treatment and gCpG based tumor vaccine thus dramatically enhanced the therapeutic efficacy of tumor growth.

## Conclusion

In present study, we chemically conjugated type B CpG with glucose and obtained a new CpG adjuvant. This adjuvant activated Th1 and Tc1, enhanced tumor-specific CTL responses and regulated TME through promoting M1 macrophages polarization and inhibiting both Treg and MDSCs.

## Data Availability

All data and materials in this study are included in the published article and its additional file.

## Abbreviations

APC: Antigen-presenting cell
DC: Dendritic cell
PRR: Pattern recognition receptor
ODN: Synthetic oligodeoxynucleotide
Th: T helper cell
Tc: Cytotoxic T cell
NK: Nature killer
MDSC: Meyloid-derived suppressor cell
OVA: Ovalbumin
MHC: Major histocompatibility complex
TME: Tumor microenvironments
TAM: Tumor associated macrophage
Treg: Regulatory T cells
PD-1: Program death-1
IFN: Interferon
